# Biochemical Response to Hyperbaric Oxygen Treatment of a Transhemispheric Penetrating Cerebral Gunshot Injury

**DOI:** 10.3389/fneur.2015.00062

**Published:** 2015-03-23

**Authors:** Eric Peter Thelin, Bo-Michael Bellander, Michael Nekludov

**Affiliations:** ^1^Section for Neurosurgery, Department of Clinical Neuroscience, Karolinska Institutet, Stockholm, Sweden; ^2^Department of Pharmacology and Physiology, Karolinska Institutet, Stockholm, Sweden

**Keywords:** hyperbaric oxygen, multi-modal monitoring, penetrating brain injury, human, microdialysis

## Abstract

Hyperbaric oxygen (HBO) therapy has been suggested a treatment option in order to reduce the development of secondary insults succeeding traumatic brain injury. This case report studied the course of a 23-year-old gentleman with a close range transhemispheric gunshot wound. The biochemical parameters, using a multi-modal monitoring in the neuro-intensive care unit, improved following HBO treatment.

## Introduction

**Figure 1:**
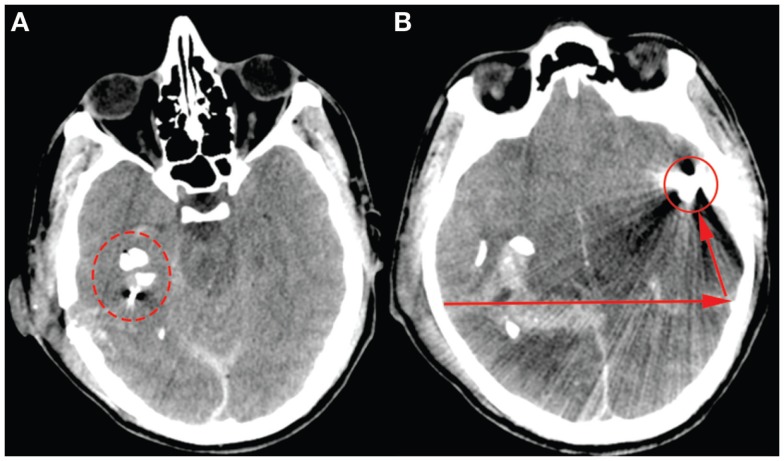
**The admission CT-scan, (A)** highlights the bone fragments close to the entry point behind the right ear (ring). **(B)** Illustrates the bullet (ring) and its trajectory (arrows).

**Figure 2:**
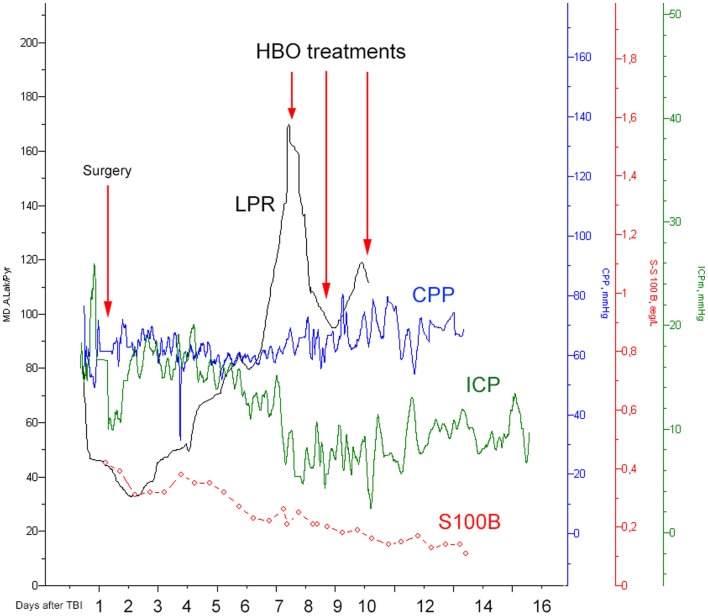
**Illustrating how the lactate:pyruvate ratio (LPR, black), the central perfusion pressure (CPP, blue), the intracranial pressure (ICP, green), and S100B (red) levels changed over time**. The first red arrow indicates the time for hemicraniectomy (“surgery”) while the other three highlight the timings for HBO treatment.

**Figure 3:**
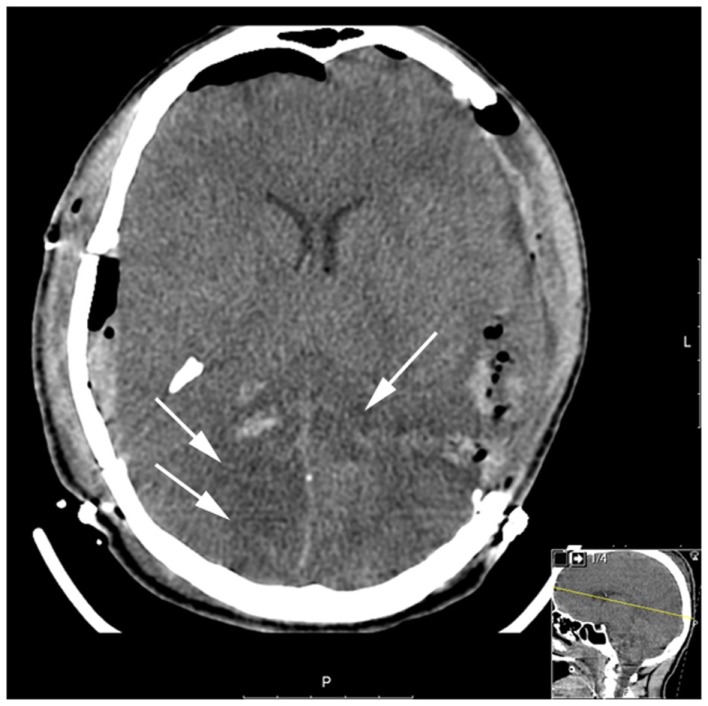
**Post-operative CT (after hemicraniectomy) day 2**. The white arrows highlight the hypodense regions of brain parenchyma in the posterior circulation which is tissue at risk for subsequent ischemic injury.

A 23-year-old male, previously healthy, was admitted to the emergency room following a cerebral gunshot wound, at close range, from a hand gun. On admission, the patient presented with a Glasgow Coma Score (GCS) of 14 (E4 + M6 + V4), obeyed commands, had normal pupil responses and extremity movement, yet was agitated and restless. Respiratory and circulatory parameters were stable. The entrance was located just behind the right ear, no exit wound was visible. Subsequently, the patient was sedated, intubated, and a full body CT-scan was performed, revealing no extracranial injuries. A CT-scan of the head revealed several bone fragments in the right temporal lobe below the entrance of the bullet (Figure 1A), which progressed through the brain with a bihemispheric central and transventricular trajectory. Along the route, intraparenchymal- and subarachnoid-hemorrhages were present (Figure 1B). The patient underwent wound revision and monitoring neurosurgery, receiving a Licox^®^ brain tissue oximetry device (PBtO2; Integra LifeSciences, Plainsboro, NJ, USA), an intracerebral microdialysis catheter (CMA70, Microdialysis AB, Stockholm, Sweden), an intracranial pressure (ICP) device (Codman^®^ DePuy Synthes, Johnson & Johnson Medical, New Brunswick, NJ, USA), and an extra-ventricular drain (Medtronic, Minneapolis, MN, USA). The patient was transferred to the neuro-intensive care unit for further treatment. During the following day, the patient deteriorated with an increased ICP, increased intracerebral lactate:pyruvate ratio (LPR), and increased serum levels of the biomarker S100B (Figure 2). This prompted an increase in administration of sedatives (propofol and midazolam), iterative doses of hypertonic saline, and infusion of pentobarbiturates. Despite this, the ICP remained elevated. The neurosurgeon on call decided to perform a hemicraniectomy in order to improve the intracranial conditions. Following the procedure, the ICP returned to normal levels (<20 mmHg), although the LPR remained high (>40) (Figure 2). The post-operative CT-scan revealed hypodense areas, indicating edema or that the tissue was at risk for ischemia, primarily in the cerebral areas supplied by the posterior circulation (Figure 3).

**Figure 4:**
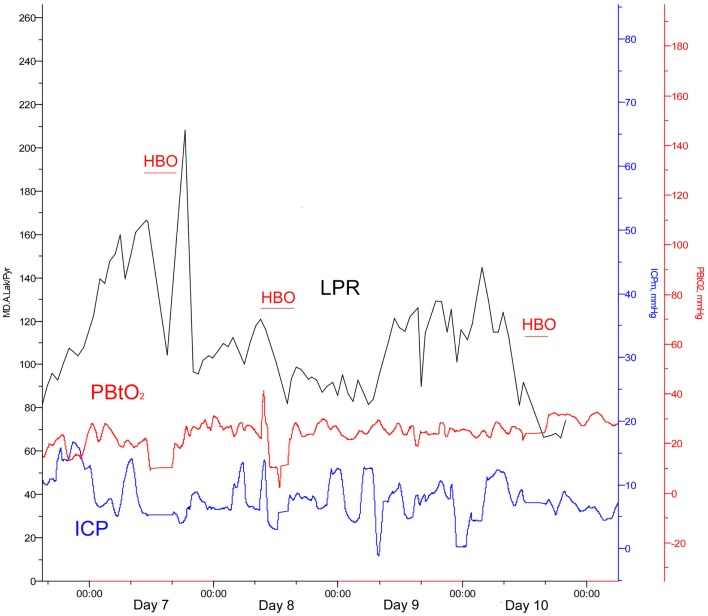
**Lactate:pyruvate ratio (LPR, black), intracranial pressure (ICP, blue), and the brain tissue oxygen pressure (PBtO2, red) changes during HBO treatment (red blocks)**.

**Figure 5:**
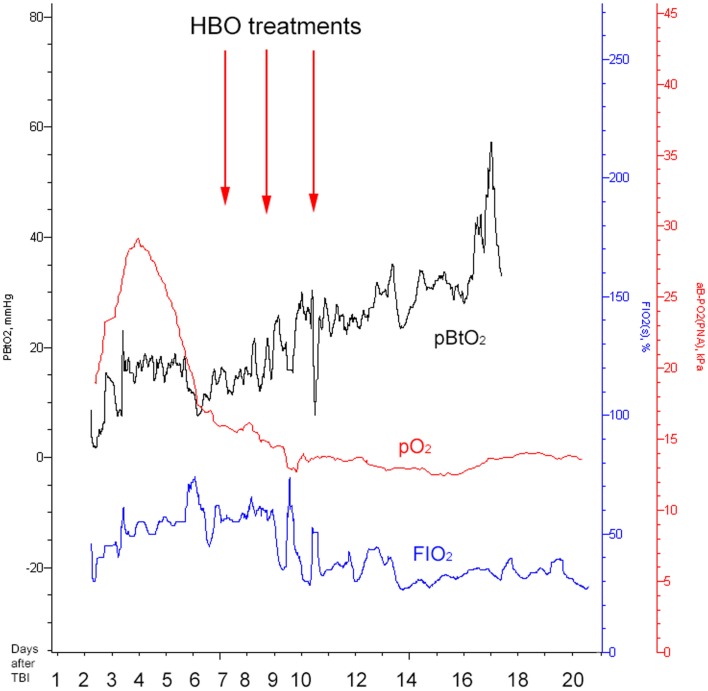
**Illustrating how the brain tissue oxygen pressure (PBtO2, black), the arterial blood gas oxygen pressure (pO2, red), and fraction of inspired oxygen (FIO2, blue) changed over time**. The red arrows indicate timing for HBO treatments. All the measurements presented are performed at normobaric conditions (before and after HBO).

On day 7 post-trauma, the patient deteriorated further, with increasing LPR and S100B levels, yet normal cerebral oxygenation (PBtO2 >15mm Hg), hence not indicating ischemia, though perhaps an ongoing mitochondrial dysfunction with a metabolic crisis. This lead to the initiation of hyperbaric oxygen (HBO) treatment (75 min, 2.8 bar with two air-brakes, followed by a stage-wise decompression during 40 min), which had an imminent, and stabilizing, effect on the LPR (Figures 2 and 4). The ICP remained normal (<20 mmHg) throughout the HBO treatment period. The PBtO2 was not monitored during the HBO, because of technical issues, but showed a sustained increase to levels above 20 mmHg after the completed hyperbaric treatment, allowing the decrease of fraction of inspired oxygen (FiO2) (Figure 5). The secondary increase of S100B stopped, and was followed by a steady decline (Figure 2). The focal LPR is measured using the microdialysis technique, which is compatible to use inside the hyperbaric chamber. However, the measured levels may not be completely reliable during the compression and decompression phases, since the speed of perfusion is influenced by the surrounding pressure changes. The patient received two further HBO treatments, same type and duration, on day 8 and day 10 post-trauma. In order to visualize the changes before and after HBO treatment, Figure 4 shows LPR, PBtO2, and ICP during the 3 days of HBO treatment. The LPR has the highest level after the HBO treatment, which is a single sample, and thus presumably false. Following this peak, there is a sustained period of lower LPR samples.

**Figure 6:**
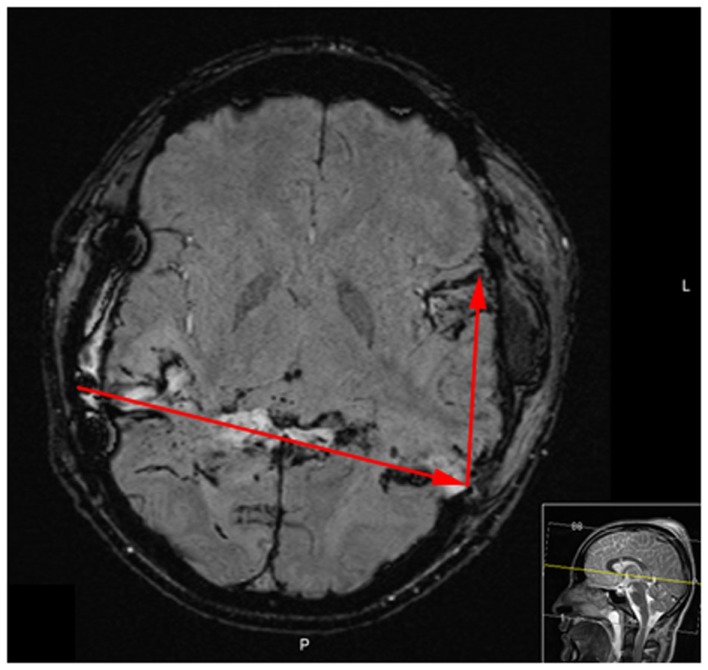
**MRI performed [susceptibility weighted imaging protocol (SWI)] on day 16 after injury reveals bilateral temporal damage and hemorrhage along the bullet, yet no signs of ischemic injury**.

Day 16 after injury, the patient was provided with a tracheostomy. A MRI on day 16 showed bilateral temporal damage with cytotoxic edema along the trajectory, yet no ischemic injury or permanent damage to the frontal lobes (Figure 6). Neurophysiological examination on day 27 revealed signs of bilateral cortical blindness (no visually evoked potentials detected), no signs of brain stem damage (normal brainstem auditory evoked potentials, BAEP), and no irregularities on the electromyogram (EMG). The patient himself was not aware of being blind.

On day 36, the patient was discharged from NICU to an intermediate neurosurgical ward.

The patient started physiotherapy and at day 38 after injury, he was able to move with support. At discharge from the neurosurgical clinic, the patient was still blind and had cognitive deficits yet had regained almost all motoric functions and could walk a shorter distance without support. Six months after trauma, the patient is still dependent on medical staff in order to move, as well as suffering from cortical blindness and cognitive shortfall.

## Background

It has been shown that patients with low GCS at admission, unresponsive pupils, bihemispheric transventricular injury, and subarachnoid hemorrhage usually have a poor outcome after cerebral gunshot wounds (1). This penetrating brain injury was treated as a severe traumatic brain injury (TBI) (GCS3–8). At our department, patients suffering from severe TBI are mechanically ventilated, and sedated with morphine, midazolam, and/or propofol. If mass lesions are present, they are evacuated as deemed appropriate. Mean arterial pressure (MAP) is measured intra-arterially. Cerebral perfusion pressure (CPP) is calculated as MAP–ICP with the transducers placed at mid-lateral ventricular level. The patients are treated in 30° sitting position. ICP is targeted at <20 mmHg (2) and CPP is targeted at 50–70 mmHg. Targets are achieved with intravenous infusions, vasopressors, osmotic therapy, and intermittent CSF drainage from ventricular catheters, ventilation, temperature control, and decompressive craniotomy, if needed. Patients are normoventilated (pCO_2_ approx. 4.5 kPa). Blood glucose is targeted at 4–8 mmol/L and hemoglobin is targeted at >90 g/L. Temperature is regulated at 37°C with paracetamol or external wrapping cooling systems. If ICP could not be regulated with the previously described techniques, sodium thiopental is infused, limited by burst suppression and monitored with continuous electroencephalography (EEG). Mild hypothermia (35–36°C) is used for high refractory ICP.

Intracerebral microdialysis is performed routinely in patients suffering from severe traumatic brain injuries (3). Increasing LPR >40 indicates tissue ischemia or mitochondrial dysfunction. We routinely obtain serum samples of the biomarker S100B every 12 h and recognize secondary peaks as a negative prognostic indicator (4). Hyperbaric treatment seemed already in the mid-1970s to improve outcome in patients suffering from TBI presenting mid-brain symptoms (5), especially during 1.5–2.0 bar for 40 min (6). Recent studies by Rockswold and co-workers have shown beneficial effect by using hyperbaric treatment with significantly improved markers of oxidative metabolism, reduced intracranial hypertension, and improvement in markers of cerebral toxicity as well as a significant reduction in mortality and improved favorable outcome (7, 8).

In the present case report, we present a patient with relatively high GCS and normal pupil response at admission, despite a gunshot wound with a detrimental bihemispheric trajectory. The patient developed an increasing LPR, indicating local tissue damage due to ischemia or mitochondrial dysfunction, and was thus treated with 2.8 bar HBO. The intracranial conditions of the patient improved, and the patient survived despite unfavorable odds.

The current setting presents a higher pressure than reported by the Rockswold group (8). This regimen was chosen as a rescue measure of “last resort,” facing the uncontrolled increase in LPR, because of presumed cerebral mitochondrial dysfunction. An increased FIO_2_ was tested, but failed to improve cerebral metabolic conditions (Figure 5). The patient’s vital and metabolic parameters were thoroughly monitored during the HBO procedure, and the pressure would be lowered if any adverse effects had been detected. Despite the success achieved in this particular case, we do not recommend this regimen as a standard HBO procedure in severe TBI cases. More studies are necessary to determine the optimal dose regimen and to individualize the treatment.

The graphs presented are exported using the LabPilot^®^ software (Microdialysis AB, Stockholm, Sweden).

## Discussion

Pathophysiology following TBI is complex, initiated by the primary damage to the brain parenchyma and a subsequent disintegration of the blood–brain barrier (BBB), leading to the development of cerebral edema. An early decrease in cerebral blood flow, hampering oxygen and substrate delivery to the cerebral tissue, leads to a subsequent development of ischemia, and eventually cellular death.

The ischemic environment present in the affected tissue, leads to an impaired mitochondrial function with ensuing anaerobic metabolism with increased intracerebral levels of lactate and increased LPR and an increased risk of development of secondary brain injuries (9, 10). However, a favorable environment in the border zone between dead and survivable tissue, may facilitate the recovery of the affected brain parenchyma, prevent negative effects of secondary insults, and by that improve patient outcome (11, 12). There are several animal models, and clinical TBI studies, elaborating the effects of normobaric hyperoxia (NBO) and HBO in TBI. Both NBO and HBO are capable of improving physiological variables, such as cerebral oxygenation (PBtO2) and metabolism (LPR) (13, 14). Experimental HBO treatments have been shown to improve mitochondrial function, increase ATP production, and reduce cell death in the hippocampus (15, 16). Recently, clinical data provided by professor Rockswold’s group confirmed beneficial effects of HBO in human severe TBI, assessed from both surrogate endpoints, such as ICP, LPR, PBtO2, but also from a significant mortality reduction and an increase of favorable outcome (8). In our case, similar to the study by Rockswold et al., the intraparenchymal LPR decreased, the PBtO2 increased, and the ICP decreased after HBO (8). Also, the HBO treatment correlated to a decrease of serum S100B levels, where secondary increases often correlate to the development of ischemic injuries, as seen in a previous study by our group (4). Despite a secondary CT-scan revealing tissue at risk for further ischemic deterioration, as well as quickly deteriorating metabolic conditions, no further ischemic, or other, injury was visible on the MRI performed on day 16.

It is important to note that the clinical course for each patient is highly individual. Even if the current HBO treatment improved conditions, its direct effect on outcome is not possible to evaluate from this particular case. The aim with multi-modal monitoring is to assess the effects of the individualized therapies provided for each patient. In this case, HBO treatment was followed by improved brain oxygenation and a better metabolic status (LPR), indicating a clinical usefulness.

The hyperbaric facility at Karolinska is equipped with a large multiplace chamber (HAUX, Germany). The working area of the chamber is 50m^2^, and the HBO department is located adjacent to the ICU. The chamber is equipped with modern ICU-hardware, similar to the ordinary ICU, but approved for use during hyperbaric conditions. This makes it possible to provide HBO treatment to patients in circulatory and respiratory distress, such as toxic shock syndrome in case of necrotizing soft tissue infections. However, patients with TBI are not routinely treated in the hyperbaric chamber at our department. In special cases, HBO treatment could be considered. Future prospective studies should be launched to further validate the effect on outcome of HBO treatment in TBI.

## Conclusion

In this case study, 2.8 bar HBO therapy improved the intracranial biochemical conditions of a patient suffering from a cerebral bihemispheric gunshot wound, indicating a potential benefit of HBO treatment. Further studies are warranted to better select which TBI patients that would best benefit from HBO treatment.

## Conflict of Interest Statement

The authors declare that the research was conducted in the absence of any commercial or financial relationships that could be construed as a potential conflict of interest.

## References

[1] Martins RS Siqueira MG Santos MT Zanon-Collange N Moraes OJ. Prognostic factors and treatment of penetrating gunshot wounds to the head. Surg Neurol (2003) 60:98–104. 10.1016/S0090-3019(03)00302-1 12900108

[2] Bratton SL Chestnut RM Ghajar J McConnell Hammond FF Harris OA Hartl R Guidelines for the management of severe traumatic brain injury. VIII. Intracranial pressure thresholds. J Neurotrauma (2007) 24:S55–8 10.1089/neu.2007.9988 17511546

[3] Bellander BM Cantais E Enblad P Hutchinson P Nordström CH Robertson C Consensus meeting on microdialysis in neurointensive care. Intensive Care Med (2004) 30:2166–9. 10.1007/s00134-004-2461-8 15549254

[4] Thelin EP Nelson DW Bellander BM. Secondary peaks of S100B in serum relate to subsequent radiological pathology in traumatic brain injury. Neurocrit Care (2014) 20:217–29. 10.1007/s12028-013-9916-0 24146416

[5] Holbach KH Wassmann H Kolberg T [Improved reversibility of the traumatic midbrain syndrome using hyperbaric oxygen]. Acta Neurochir (1974) 30:247–56 10.1007/BF01405583 4432786

[6] Holbach KH Caroli A Wassmann H Cerebral energy metabolism in patients with brain lesions of normo- and hyperbaric oxygen pressures. J Neurol (1977) 217:17–30 10.1007/BF00316313 75249

[7] Rockswold SB Rockswold GL Zaun DA Zhang X Cerra CE Bergman TA A prospective, randomized clinical trial to compare the effect of hyperbaric to normobaric hyperoxia on cerebral metabolism, intracranial pressure, and oxygen toxicity in severe traumatic brain injury. J Neurosurg (2010) 112:1080–94. 10.3171/2009.7.JNS09363 19852540

[8] Rockswold SB Rockswold GL Zaun DA Liu J. A prospective, randomized Phase II clinical trial to evaluate the effect of combined hyperbaric and normobaric hyperoxia on cerebral metabolism, intracranial pressure, oxygen toxicity, and clinical outcome in severe traumatic brain injury. J Neurosurg (2013) 118:1317–28. 10.3171/2013.2.JNS121468 23510092 PMC12928550

[9] Chesnut RM Marshall LF Klauber MR Blunt BA Baldwin N Eisenberg HM The role of secondary brain injury in determining outcome from severe head injury. J Trauma (1993) 34:216–22. 10.1097/00005373-199302000-00006 8459458

[10] Enriquez P Bullock R. Molecular and cellular mechanisms in the pathophysiology of severe head injury. Curr Pharm Des (2004) 10:2131–43. 10.2174/1381612043384060 15281889

[11] Bratton SL Chestnut RM Ghajar J McConnell Hammond FF Harris OA Hartl R Guidelines for the management of severe traumatic brain injury. I. Blood pressure and oxygenation. J Neurotrauma (2007) 24:S7–13 10.1089/neu.2007.9995 17511549

[12] Werner C Engelhard K Pathophysiology of traumatic brain injury. Br J Anaesth (2007) 99:4–9 10.1093/bja/aem131 17573392

[13] Menzel M Doppenberg EM Zauner A Soukup J Reinert MM Bullock R. Increased inspired oxygen concentration as a factor in improved brain tissue oxygenation and tissue lactate levels after severe human head injury. J Neurosurg (1999) 91:1–10. 10.3171/jns.1999.91.1.0001 10389873

[14] Beynon C Kiening KL Orakcioglu B Unterberg AW Sakowitz OW. Brain tissue oxygen monitoring and hyperoxic treatment in patients with traumatic brain injury. J Neurotrauma (2012) 29:2109–23. 10.1089/neu.2012.2365 22616852

[15] Daugherty WP Levasseur JE Sun D Rockswold GL Bullock MR Effects of hyperbaric oxygen therapy on cerebral oxygenation and mitochondrial function following moderate lateral fluid-percussion injury in rats. J Neurosurg (2004) 101:499–504 10.3171/jns.2004.101.3.0499 15352608

[16] Zhou Z Daugherty WP Sun D Levasseur JE Altememi N Hamm RJ Protection of mitochondrial function and improvement in cognitive recovery in rats treated with hyperbaric oxygen following lateral fluid-percussion injury. J Neurosurg (2007) 106:687–94. 10.3171/jns.2007.106.4.687 17432723

